# Negated bio-events: analysis and identification

**DOI:** 10.1186/1471-2105-14-14

**Published:** 2013-01-16

**Authors:** Raheel Nawaz, Paul Thompson, Sophia Ananiadou

**Affiliations:** 1 National Centre for Text Mining, Manchester Interdisciplinary Biocentre, University of Manchester, 131 Princess Street, Manchester M1 7DN, UK

## Abstract

**Background:**

Negation occurs frequently in scientific literature, especially in biomedical literature. It has previously been reported that around 13% of sentences found in biomedical research articles contain negation. Historically, the main motivation for identifying negated events has been to ensure their exclusion from lists of extracted interactions. However, recently, there has been a growing interest in negative results, which has resulted in negation detection being identified as a key challenge in biomedical relation extraction. In this article, we focus on the problem of identifying negated bio-events, given gold standard event annotations.

**Results:**

We have conducted a detailed analysis of three open access bio-event corpora containing negation information (i.e., GENIA Event, BioInfer and BioNLP’09 ST), and have identified the main types of negated bio-events. We have analysed the key aspects of a machine learning solution to the problem of detecting negated events, including selection of negation cues, feature engineering and the choice of learning algorithm. Combining the best solutions for each aspect of the problem, we propose a novel framework for the identification of negated bio-events. We have evaluated our system on each of the three open access corpora mentioned above. The performance of the system significantly surpasses the best results previously reported on the BioNLP’09 ST corpus, and achieves even better results on the GENIA Event and BioInfer corpora, both of which contain more varied and complex events.

**Conclusions:**

Recently, in the field of biomedical text mining, the development and enhancement of event-based systems has received significant interest. The ability to identify negated events is a key performance element for these systems. We have conducted the first detailed study on the analysis and identification of negated bio-events. Our proposed framework can be integrated with state-of-the-art event extraction systems. The resulting systems will be able to extract bio-events with attached polarities from textual documents, which can serve as the foundation for more elaborate systems that are able to detect mutually contradicting bio-events.

## Background

### Introduction

Owing to the rapid advances in biomedical research, scientific literature is being published at an ever-increasing rate [1]. For example, the size of PubMed is increasing at the rate of approximately two papers per minute [2]. As a result, it is becoming increasingly difficult for biologists to keep abreast of developments within biomedicine, and automated means are required to satisfy their information needs. Consequently, text mining is receiving increasing interest within the biomedical field [3-5], as it enriches text via the addition of semantic metadata, and thus permits tasks such as analysing molecular pathways [6] and performing semantic searches [7].

#### Event-based text mining

Event-based text mining approaches constitute a promising alternative to the traditional approaches, which are mainly based on the bag-of-words principle [7-9]. Textual events are template-like, structured representations of pieces of knowledge contained within documents. Text mining systems that are able to extract events automatically can allow much more precise and focussed retrieval and extraction of information than the traditional keyword-based systems [8]. Event-based retrieval allows the user to specify one or more constraints on the events to be retrieved, without having to be concerned about the precise wording used in the text. These constraints could be in terms of the type of the event, and/or the type(s) of its participants, and/or the precise format of participants playing particular roles in the event. An example of such a system is MEDIE [7], a semantic search engine for MEDLINE [10] abstracts.

Furthermore, the event representation of text allows a document to be viewed as a collection of nested textual events. We call this the **event view** of a document. When used in conjunction with event and term ontologies, the event view can facilitate the extraction of the implicit relations present in the text. Therefore, the events extracted from a document can be used to facilitate more challenging text mining tasks like recognition of textual inference, i.e., detection of entailment and contradiction in textual sources [11].

#### Significance of detecting negated bio-events

Negation is considered a universal property of all human languages [12]. However, the concept and manifestation of negation in natural languages is far more subtle and complex in force and scope than it is in formal logic [13-15]. Nonetheless, negation occurs frequently in scientific literature, especially in the domain of biomedicine. Vincze et al. [16] report that around 13% of sentences found in biomedical research articles contain some form of negation. Our analysis of three open access bio-event corpora showed that more than 6% of bio-events are negated.

Historically, in the field of biomedical text mining, the main motivation for the identification of negated events has been to ensure their exclusion from an extracted list of interactions. This was mainly because most biomedical research has been focussed around the publication and analysis of positive results [17]. However, over the past decade, there has been a growing interest in negative results, for example:

• The Journal of Negative Results in Biomedicine [18] has been launched, which, as the name suggests, focusses specifically on negative results.

• The Negatome database [19] has been released, which provides information about non-interacting protein pairs.

• Efforts have been made to incorporate negation in the popular biomedical ontologies [20].

Furthermore, negation detection has been identified as the foremost challenge in biomedical relation extraction [21]. More specifically, it has been argued that the recognition of negated bio-events is of fundamental practical significance for researchers in most biomedical disciplines [22].

In response to the importance of detecting negated bio-events, we have carried out an in-depth analysis of three open access bio-event corpora containing negation information, and propose a new classification of negated bio-events. We have subsequently used the information resulting from our analysis to feed into the design of a machine learning solution to the problem of detecting negated bio-events. The resulting novel framework for the identification of negated bio-events has been evaluated on each of the three open access corpora mentioned above, achieving significantly better results than the existing state-of-the-art systems.

### The task of identifying negated bio-events

This section provides a brief overview of bio-events and describes the problem of identifying negated bio-events.

#### Bio-events

In its most general form, a **textual event** is as an action, relation, process or state expressed in the text [23]. More specifically, a textual event is a structured semantic representation of a certain piece of information contained within the text. Textual events are usually anchored to particular text fragments that are central to the description of the event. The most important of these text fragments is the *event-trigger*, which is usually a verb or a noun that indicates the occurrence of the event. Events are often represented as a template-like structure with slots that are filled by the event *participants*. These event participants describe the different aspects of the event, e.g., what causes the event, what is affected by it, where it took place, etc. Based on its function, each participant is usually assigned a role within the event. The participants can correspond to entities, concepts or even other events. If an event contains other events as its participants, then it is called a *complex* event. This kind of event representation allows the information contained within a piece of text to be represented as a collection of nested events.

A **bio-event** is a textual event specialised for the biomedical domain. Kim et al. [9] define a bio-event as a dynamic bio-relation involving one or more participants. These participants can be bio-entities or (other) bio-events, and are usually each assigned a semantic role/slot like *theme* and *cause*, etc. Each bio-event is typically assigned a type/class from a chosen bio-event taxonomy/ontology, e.g., the GENIA Event Ontology [9]. Similarly, the bio-entities are normally also assigned types/classes from a chosen taxonomy/ontology, e.g., the Gene Ontology [24]. The template of a bio-event can also contain additional slots, e.g., to denote temporal and spatial attributes.

**Figure 1:**
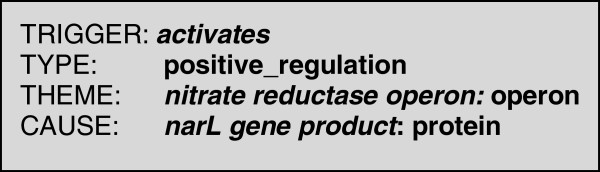
Typical structured representation of the bio-event contained in the above sentence.

As an example, consider the following sentence from the GENIA Event corpus (PMID: 3035558): “*The results suggest that the narL gene product activates the nitrate reductase operon”.* According to the GENIA Event annotation scheme, this sentence contains a single bio-event, anchored to the verb *activates*. Figure 1 shows the structured representation of this bio-event.

The fact that the event is anchored to the word *activates* allows the event-type of *positive_regulation* to be assigned. The event has two slots, i.e., *theme* and *cause,* whose labels help to characterise the contribution that the slot filler makes towards the meaning of the event. In this case, the slots are filled by the subject and object of the verb *activate*, both of which correspond to different types of bio-entities (i.e., *operon* and *protein).*

**Figure 2:**
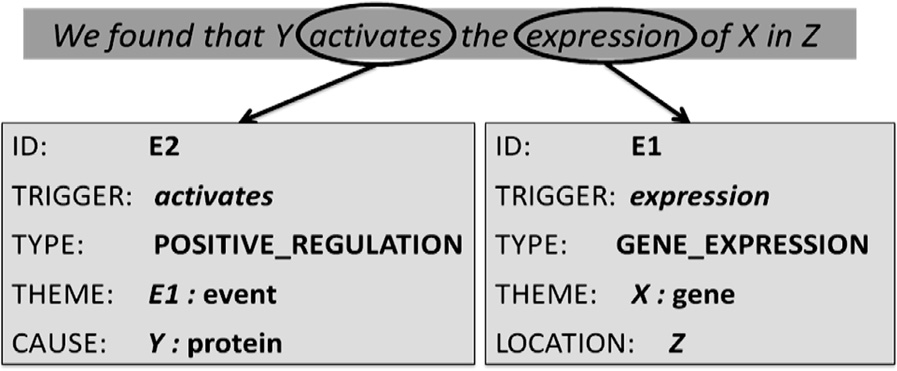
A simple hypothetical sentence with complex event structure.

Figure 2 shows a simple hypothetical sentence with a more complex event structure. The event E1 is anchored to the word *expression* and has been assigned the event-type of *gene_expression*. It has a single participant, the arbitrary gene *X*, which acts as the theme of the event. E1 also has a location attribute, which has the arbitrary value of *Z*. The word *activates* has been identified as the event-trigger for the complex event E2, which has been classed as a *positive_regulation* event. It has two participants: the arbitrary protein *Y* and the event E1, which act as the cause and the theme of the event, respectively.

#### Bio-event corpora for training and evaluation

Recently, significant effort has been put into the creation of various bio-event corpora. Although each of these corpora has been created with different aims and motivations, they all contain bio-events of varying levels of generality. Furthermore, while the general definition of bio-events in all corpora complies with the description above, the exact specification of a bio-event, the types of participants and the semantic roles ascribed to them vary from corpus to corpus. A brief description of some of these corpora is as follows:

• GENIA Event: The GENIA Event corpus [9] contains 1,000 MEDLINE abstracts in which 36,858 bio-events have been identified. Each event belongs to one of the 36 event classes defined in the GENIA Event Ontology. The event participants can be bio-entities or other bio-events. Each bio-entity belongs to one of the 46 classes defined in the GENIA Term Ontology. Other than the participants, an event may contain additional attributes including location, time and experimental context.

• BioInfer: The BioInfer corpus [25] contains 1,100 sentences in which 2,662 bio-events have been identified. Each event belongs to one of the 60 event classes defined in the BioInfer Relationship Ontology. It is important to note that a more general definition of bio-event has been used in BioInfer, in that static bio-relations [26] have also been marked as bio-events.

• GREC: The Gene Regulation Event Corpus (GREC) [27] contains 240 MEDLINE abstracts in which 3,067 bio-events have been identified. Each event has a set of arguments, which can include both the event participants and attributes like time, location and manner, etc.

• GeneReg: The GeneReg [28] corpus contains 314 MEDLINE abstracts in which 1,770 bio-events have been identified. Each event belongs to one of the 4 classes from the Gene Regulation Ontology.

• BioNLP’09 ST (Shared Task): The BioNLP’09 ST Corpus [29] contains 950 MEDLINE abstracts. This corpus contains two subsets: the *Development* subset, comprising 150 abstracts and the *Training* subset, comprising 800 abstracts. The corpus contains a total of 11,480 bio-events, and each bio-event belongs to one of 9 event classes, which form a subset of the classes in the GENIA Event Ontology.

#### Negated bio-events

Vincze et al. [16] define negation in the context of biomedical literature as ‘the implication of the non-existence of something’. Negation at the bio-event level is non-existence of an event, i.e., a negated bio-event indicates the non-existence of that event. The indication of non-existence could be explicit (e.g., the presence of a negation marker) or implicit (e.g., semantic inference).

Amongst the bio-event corpora introduced above, only three contain annotations relating to event polarity, i.e., GENIA Event, BioInfer and BioNLP’09 ST. Negation cues (i.e., words and phrases that explicitly indicate a negation) have been explicitly annotated only in BioInfer. Table 1 shows statistics regarding the annotations present in each of the three corpora. In terms of volume, the GENIA Event corpus is the largest, with almost 37,000 events, while BioInfer is the smallest, with fewer than 2,700 bio-events. Regarding event-types, BioInfer is the richest, with 60 event-types, whilst BioNLP’09 ST is the simplest, with only 9 event-types. Interestingly, the distribution of negated bio-events in all three corpora is fairly uniform, ranging between 6.1% and 6.4%.

**Table 1:** Statistics of bio-event corpora containing polarity information

**Corpus**	**Event types**	**Total events**	**Negated events**	**Negation percentage**
GENIA Event	36	36,858	2,351	6.4%
BioInfer	60	2,662	163	6.1%
BioNLP’09 Shared Task	9	11,480	722	6.3%

#### Identification of negated bio-events: task description and analysis

Following previous work [29-33], we treat the task of identifying negated bio-events as an independent task in itself. That is, we assume that event annotation has already been performed, and aim to create an automated means of classifying the identified events according to their polarity.

A related negation detection task, which has recently received significant attention, is the detection of negation scopes [34]. This involves the identification of the sequence of words within in a sentence that is affected by a particular negation cue. Despite the apparent similarities, identification of negated bio-events is essentially different from negation scope detection. While scope annotation focusses on linguistic properties of the text, the goal of bio-event annotation is to identify which kinds of biological information appear in which parts of text. Therefore, the identification of bio-events in text has two distinguishing characteristics [9,25,27]:

1. Bio-event annotation is information-centred and depends entirely on biologists’ conception of the relationship between an event, its participants and other events expressed in the text.

2. The event-trigger and participants of an event are each mapped to a span of text. This usually causes the description of an event to be spread over several discontinuous spans in text, which could belong to different clauses within a sentence.

In contrast to the above characteristics, the scopes of negation cues are continuous and relatively less ambiguous [16]. A few interesting consequences of this contrast are:

• A sentence containing a negation cue may not contain any negated events at all.

• At the other extreme, negated events may be present even when a negation cue is not present in the sentence.

• The event-triggers and/or participants of several events may fall under the scope of a negation cue. However, it is highly unlikely that all of these events will be negated.

Vincze et al. [35] conducted an in-depth comparison of a linguistically annotated corpus of negation scopes (BioScope) and a biologically annotated corpus of negated bio-events (GENIA Event). They found that only half (51%) of the bio-events with event-triggers inside the scope of a negation cue were actually negated. Conversely, 16% of negated bio-events had event-triggers which were outside the scope of the negation cues present in the sentence containing the event. They concluded that negation scope detection is not sufficient for the identification of negated bio-events, as the latter is a more complex task, which requires a deeper and more complex analysis than other negation detection tasks, like negated term detection and negation scope detection.

### Related work

This section provides a brief overview of the previous work done on types of negation, negation cues, detection of negated terms and negation scopes, detection of negated protein-protein interactions (PPIs) and identification of negated bio-events.

#### Types of negation

One of the first attempts at classifying negation in natural language was made by Aristotle. He concluded that negations can be divided into four types, which he named as correlation (e.g., *double* vs. *half*), contrariety (e.g., *good* vs. *bad*), privation (e.g., *blind* vs. *sighted*) and contradiction (e.g., *he sits* vs. *he does not sit*) [14]. In terms of more recent work, Tottie [13] presented a taxonomy of clausal negations in English. She identified 6 top-level categories of clausal negation: denials, rejections, imperatives, questions, supports and repetitions. Harabagiu et al. [36] identified two main classes of negation: directly licensed negations and indirectly licensed negations. The directly licensed negations include: overt negative markers (such as *not*), negative quantifiers (like *no*) and strongly negative adverbs (like *never*). The indirectly licensed negations include: verbs or phrasal verbs (such as *fail*), prepositions (such as *without*), weak quantifiers (such as *few*) and traditional negative polarity items (such as *a red cent*). Huang and Lowe [37] proposed a classification of negations found in medical reports. Their classification was based on the syntactic category of the negation signal and phrase patterns. They identified 4 syntactic categories of negation signals: adjective-like (such as *no*, *absent* and *without*), adverb (such as *not*), verb (such as *deny*) and noun (such as *absence*). They also identified 9 phrase patterns corresponding to the syntactic categories.

To our knowledge, the only previous study on the classification of negated bio-events was reported by Sanchez-Graillet and Poesio [38], who analysed negated PPIs in 50 biomedical articles. They identified seven classes of negation for PPIs, based on lexical and syntactic patterns.

The different studies outlined above suggest that the best way to classify negations appears to be domain-specific. Although the work of Sanchez-Graillet and Poesio concerns bio-events, their classification is specific to PPIs and cannot be trivially extended to all types of bio-events. Therefore, a more general framework is required, which can be applied to classify all types negated bio-events.

#### Negation cues

A number of different studies have identified negation cues that appear in medical and biomedical texts. Chapman et al. [39] compiled a comprehensive list of 272 negation cues specific to medical discharge summaries. They reported that two negation cues (*no* and *without*) accounted for a large proportion of negative statements. Their results showed that the distribution of negation cues is Zipfian in nature. Similar results were also reported by Mutalik et al. [15], who, despite identifying over 60 negation cues, found that only a small set of cues account for the majority of negation instances. In their corpus of 40 medical documents, only four negation cues accounted for almost 93% of all negation instances. These cues are *no* (49%), *denies/denied* (21%), *not* (13%) and *without* (10%). A further study by Tolentino et al. [40] analysed negated biomedical concepts occurring in a corpus of 41 medical documents. They found that only 5 negation cues (*no*, *neither/nor*, *ruled out*, *denies* and *without*) accounted for 89% of all negated concepts found in the corpus.

Elkin et al. [41] created an ontology of terms that start negations (e.g., *no*, *denies* and *ruled out*) and another set which stop the propagation of the assignment of negation (e.g., *other than*). Kilicoglu and Bergler [30] created a list of 9 negation cues from the BioNLP’09 ST corpus. Morante [42] compiled a list of negation cues observed in the BioScope [16] corpus, identifying 8 ambiguous and 21 unambiguous negation cues. She also provided a description for the scope of each cue based on its syntactic context. Sarafraz and Nenadic [33] used the previous studies on negation to derive a primary list of 14 negation cues. They further compiled a secondary list of 18 additional negation cues that were semi-automatically extracted from the BioNLP’09 ST corpus. Interestingly, their list contains the word *inhibit*, which is treated as an indicator of *negative_regulation* (i.e., a positive event indicating down-regulation) rather than a marker of negation in the BioNLP’09 ST, GENIA Event and BioInfer corpora.

In terms of automated approaches, Morante and Daelemans [43] proposed a machine learning system for the identification of negation cues. Their system achieved an F-score of over 99% for both clinical notes and biomedical abstracts. However, their system treated 17 strings as unambiguous negation markers, i.e., every occurrence of these strings was treated as a negation cue. These unambiguous cues accounted for 95% of all instances of negations. Agarwal and Yu [44] developed a system for the automatic identification of negation cues using Conditional Random Fields (CRF). Their system achieved an F-score of 98% for clinical notes and 97% for biomedical abstracts.

The numbers of negation cues identified in the above studies vary considerably. It appears that the optimal negation cue list varies, both according to the domain of the text and the exact context/task in which they are identified.

#### Detection of negated terms, negated sentences and negation scopes

The bulk of work on negation detection in the biomedical domain has been focussed on the detection of negated terms in medical reports. This work includes both rule-based and machine learning approaches. The key rule-based solutions include those presented by Chapman et al. [39], Mutalik et al. [15], Elkin et al. [41], Huang and Lowe [37] and Boytcheva et al. [45]. The key machine learning approaches include the systems presented by Averbuch et al. [46], Goldin and Chapman [47], Goryachev et al. [48], Rokach et al. [49] and Councill et al. [50].

Wilbur et al. [51] created a corpus of 6,945 text fragments (sentences and clauses) in which each fragment is annotated along five dimensions, one of which is polarity. Their corpus contained 6,498 (94%) positive and 447 (6%) negative fragments. Shatkay et al. [52] used this corpus to develop an automated system for identifying negated text fragments. Their system achieved a precision of 96% and a recall of 93%.

Vincze et al. [16] developed BioScope, an open access corpus of biomedical text containing token level annotations for negation cues and their respective scopes. The BioScope corpus comprises three sub-corpora: (1) clinical reports containing 6,383 sentences, (2) biomedical articles containing 2,670 sentences and (3) biomedical abstracts containing 11,871 sentences. Morante and Daelemans [43] presented a machine learning approach to detecting the scope of negation cues and tested their system on the BioScope corpus. Their system determined the full scope of negation cues with an accuracy of 66% for abstracts, 41% for papers and 71% for clinical notes.

#### Detection of negated PPIs

Sanchez-Graillet and Poesio [38] developed a set of heuristics for extracting negated PPIs from biomedical articles. They implemented their system using a Functional Dependency Grammar (FDG) parser. Their preliminary results ranged from 54% to 63% F-score, depending on the method of protein name recognition. The system achieved 77% F-score when used with gold standard protein annotations.

#### Detection of negated bio-events

Identification of negated bio-events was an optional sub-task in the BioNLP’09 Shared Task Challenge [29]. Six teams participated in this task and reported the first results on the identification of negated bio-events. The rule-based system of Kilicoglu and Bergler [30] was ranked in first position, with 14% recall, 51% precision and 23% F-score. Van Landeghem et al. [32] achieved the second best results of 11% recall, 45% precision and 17% F-score. They also used a customised rule-based system. MacKinlay et al. [31] used a machine-learning approach with complex deep parse features. Their system achieved the third best results with 5% recall, 34% precision and 9% F-score. It is important to note that these systems did not operate on gold standard event annotations. Instead, they performed event extraction as a preliminary step to the identification of negated events. The approximated F-scores for these systems if gold standard event annotations were provided are 38%, 26% and 28%, respectively. These values have been calculated using a linear extrapolation function and the maximum (100%) recall value for event extraction.

Sarafraz and Nenadic [33] proposed a machine learning approach for the identification of negated bio-events. They implemented an SVM classifier with a linear kernel using features engineered from a sentence parse tree with lexical cues. They trained their classifier on the BioNLP’09 training dataset and tested it on the BioNLP’09 development dataset. They achieved 38% precision, 76% recall and 51% F-score. In a further experiment, they split the data into smaller sets containing different event-types, and trained and tested the classifier separately for each smaller dataset. This way, they achieved a micro average of 49% precision, 88% recall and 63% F-score.

## Methods

### Types of negated bio-events

We conducted an in-depth analysis of the types of negation observed in the three open access bio-event corpora containing negation annotation. We analysed a total of 1,000 randomly selected negated bio-events, of which 600 were from the GENIA Event corpus (over 25% of all negated events in the corpus), 300 were from the BioNLP’09 Shared Task corpus (over 40% of all negated bio-events in the corpus) and 100 were from the BioInfer corpus (over 60% of all the negated bio-events in the corpus). Our analysis revealed five main types of negated bio-events:

#### Inherently negative bio-events

**Figure 3:**
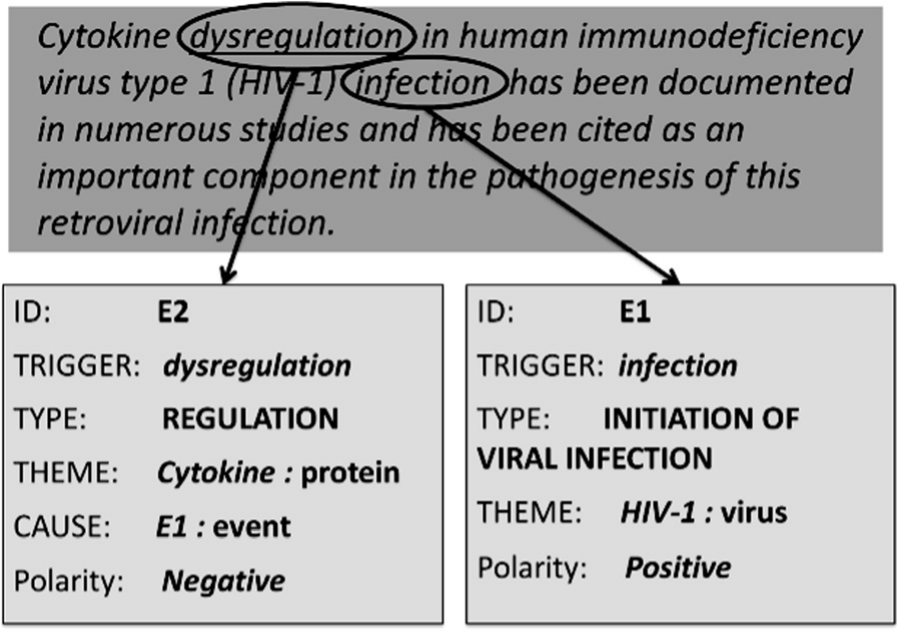
Inherently negative bio-event example (Source: GENIA Event Corpus; PMID: 9427533).

These are bio-events in which the event-trigger is itself a negation cue, like *independent*, *immobilization*, *unaffected*, *dysregulation*, etc. As an example, consider the sentence shown in Figure 3. The event E1 is triggered by the word *infection* and represents the initiation of viral infection of *HIV-1*. The event E2 is triggered by the word *dysregulation* and expresses the non-existence of the *regulation* of *Cytokine* caused by E1; therefore it has been annotated as a negated event.

#### Negated event-trigger

**Figure 4:**
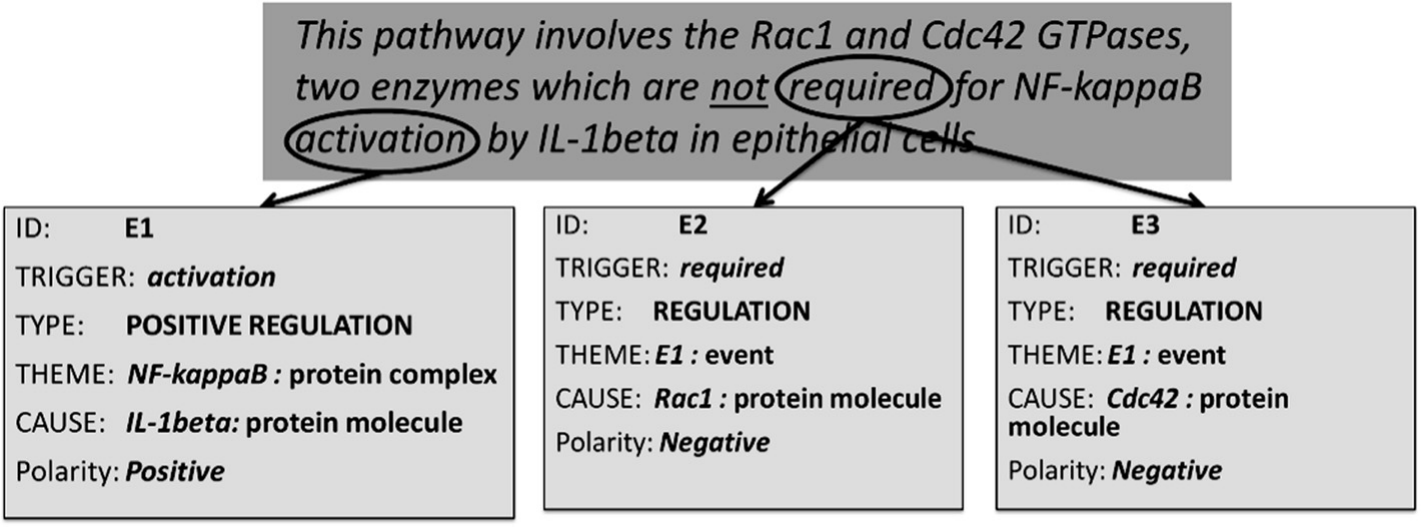
Bio-event example with negated event-trigger (Source: GENIA Event Corpus; PMID: 10022882).

In this category, an explicit negation cue modifies the event-trigger. For example, consider the sentence shown in Figure 4. The event E1 indicates the *positive regulation* of *NF-KappaB* by *IL-1beta*, where the events E2 and E3 indicate the *regulation* of E1 by the *GTPases* (protein molecules) *Rac1* and *Cdc42*, respectively. Both E2 and E3 are negated, as they are both triggered by the word *required*, which is being modified by the explicit negation cue *not*. Interestingly, the scope of the negation cue (*not*), according to the BioScope annotation guidelines, also includes the trigger for event E1 (which is not negated).

#### Negated participant

**Figure 5:**
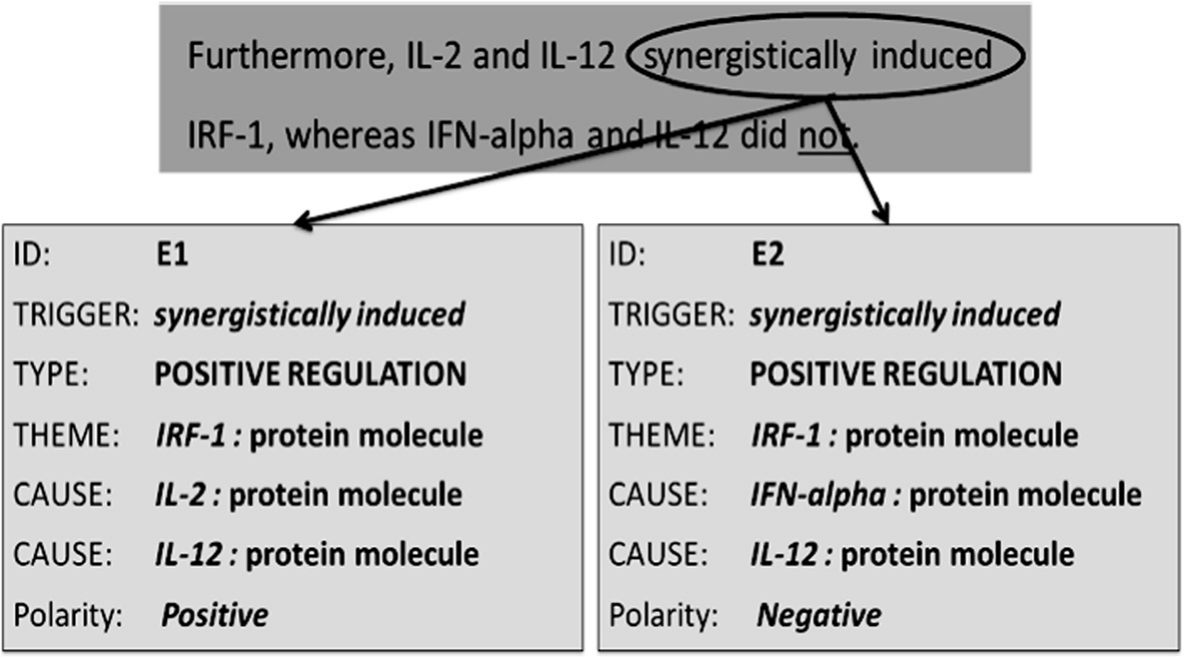
Bio-event example with negated participant (Source: GENIA Event Corpus; PMID: 10358173).

This category corresponds to bio-events in which at least one participant (theme or cause) is modified by an explicit negation cue. As an example, consider the sentence shown in Figure 5. Both events, E1 and E2, are triggered by the phrase *synergistically induced*; however, they have opposite polarities. Event E1 expresses the *positive_regulation* of *IRF-1* by *IL-2* and *IL-12*, while E2 expresses the non-existence of *positive_regulation* of *IRF-1* by *IFN-alpha* and *IL-12*. The explicit negation cue *not* modifies the two causes of E2, i.e., *IFN-alpha* and *IL-12*. Again, it is important to note that the scope of this cue (*not*) includes neither the trigger for event E2 nor any of its participants.

#### Negated attribute

**Figure 6:**
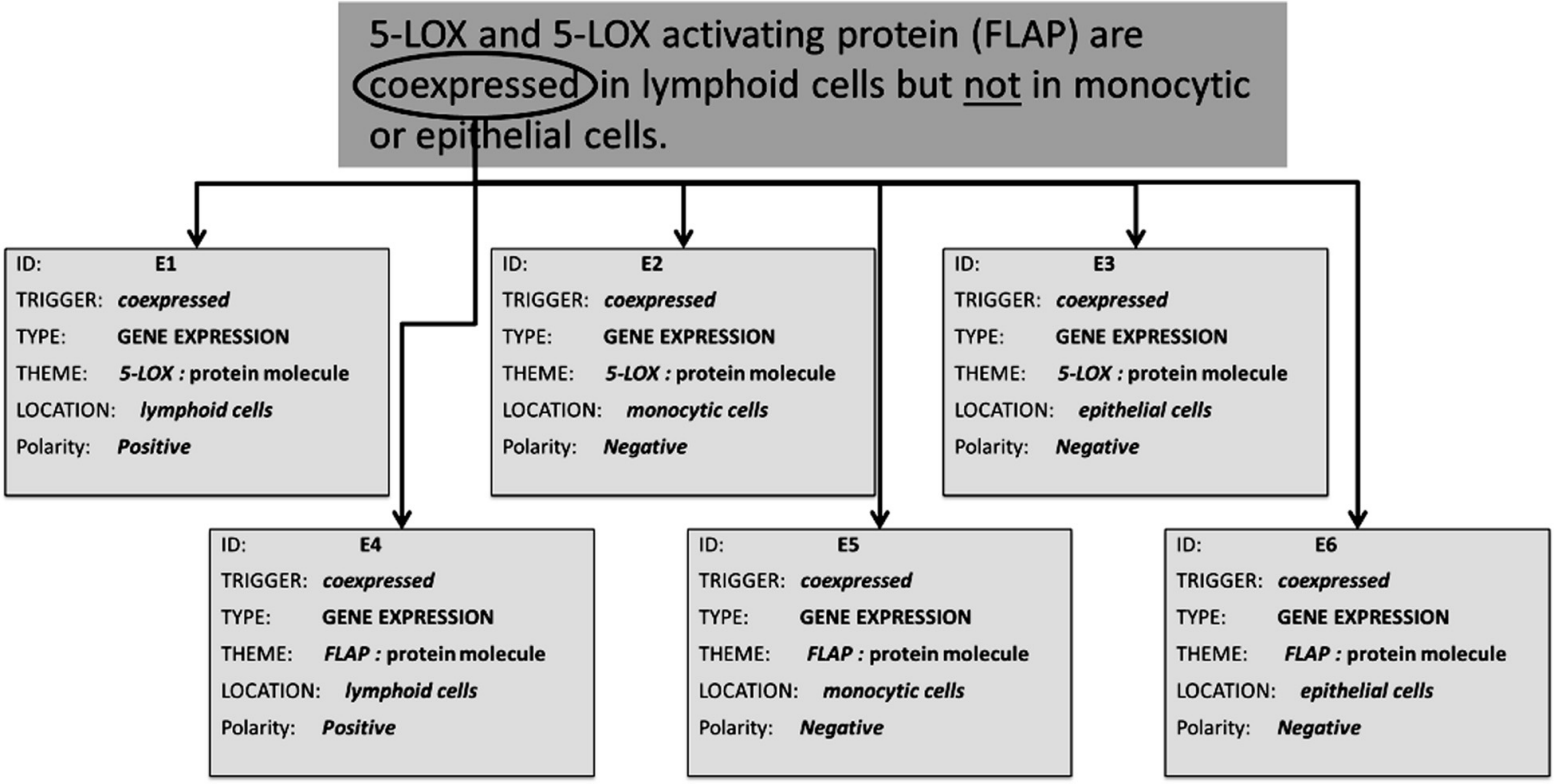
Bio-event example with negated attribute (Source: BioNLP ST Corpus; PMID: 10022882).

There are bio-events in which an explicit negation cue modifies an event attribute, such as location. An example of this type of negation is shown in Figure 6. The events E1, E2, E3, E4, E5 and E6 are all triggered by the word *coexpressed*. However, E1 and E4 represent the *expression* of the genes *5-LOX* and *FLAP* (respectively) in *lymphoid cells*, while E2, E3, E5 and E6 represent the *expression* of these genes in *monocytic* and *epithelial cells* respectively. The explicit negation cue *not* modifies the phrase *in monocytic or epithelial cells*. This phrase contains the location for E2, E3, E5 and E6, making these events negated.

Despite its relatively low frequency, this is an important type of negated bio-event. In a recent article on biologists’ perspective of negation, Krallinger [22] identified events with negated locations as of particular interest to biomedical practitioners.

#### Comparison and contrast

**Figure 7:**
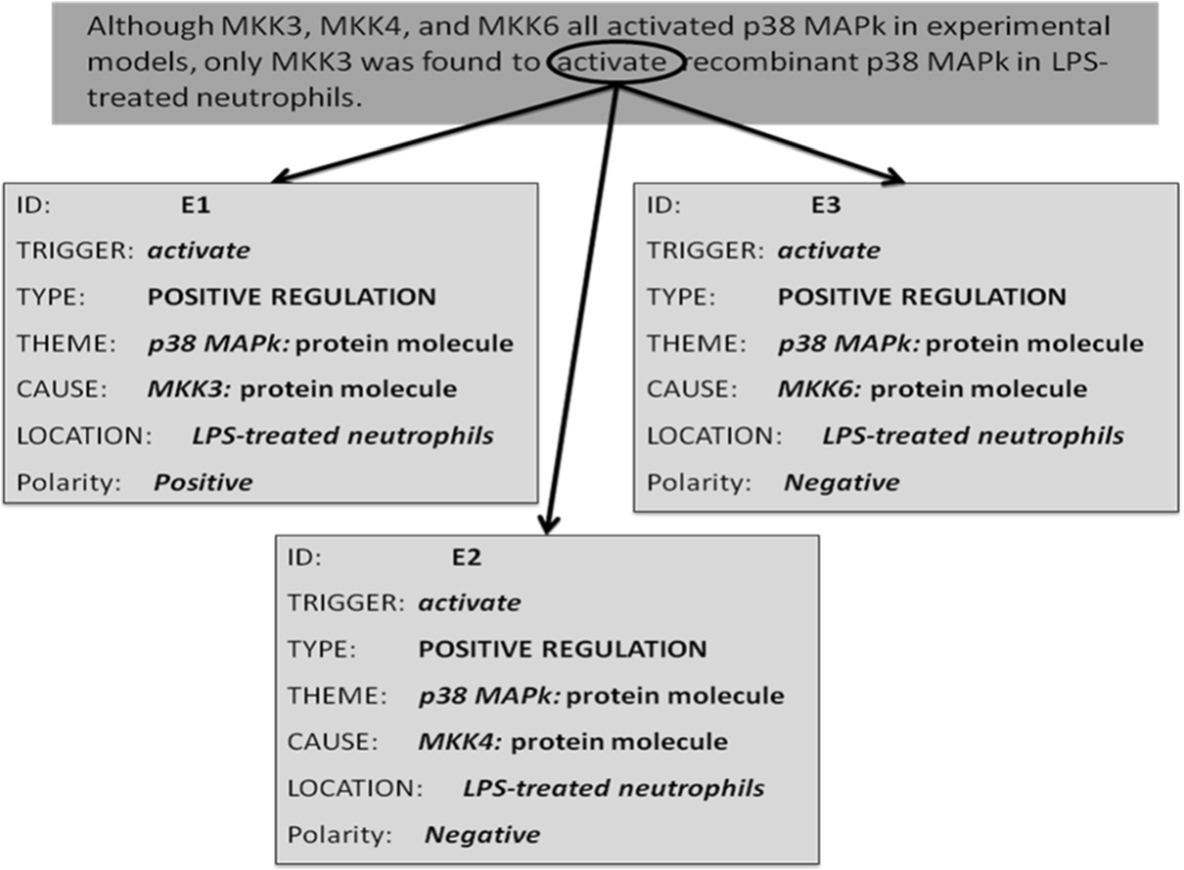
Bio-event example with comparison and contrast (Source: GENIA Event Corpus; PMID: 10079106).

This category corresponds to bio-events where the negation is signalled via contrast or comparison, normally with another bio-event. Sentences containing such negated events often lack an explicit negation marker. However, the BioInfer corpus is unique in the sense that it annotates even contrast and comparison markers as negation cues. Figure 7 shows an example of this type of negation. The event E1 is triggered by the word *activate*, and it expresses the *positive_regulation* of *p38 MAPk* by *MKK3* in *LPS-treated neutrophils*. The events E2 and E3 are similar to E1 except that they are caused by *MKK4* and *MKK6*, respectively. Both E2 and E3 have been annotated as negated; this is despite the fact that the sentence lacks an explicit negation cue.

#### Distribution

Our analysis revealed that the instances of each of the five main types of negated bio-events are present in the three corpora with varying frequencies. Table 2 shows the distributions in the three different corpora and the macro and micro averages for each type.

**Table 2:** Corpus-wise class distribution of negated bio-events

**Type**	**GENIA Event**	**BioInfer**	**BioNLP’09 ST**	**Macro average**	**Micro average**
Inherently Negative	13%	11%	9%	11%	12%
Negated Trigger	61%	62%	67%	63%	63%
Negated Participant	10%	17%	12%	14%	11%
Negated Attribute	7%	2%	6%	4%	6%
Comparison and Contrast	9%	8%	6%	8%	8%

The frequency of *inherently negative* bio-events ranges between 9% and 13%, with a micro average of 12%. This is the second most prevalent type in GENIA Event and the third most prevalent in BioInfer and BioNLP’09 ST. The frequency of *negated trigger* events ranges between 61% and 67% in the three corpora, with a micro average of 63%. This is the predominant type in all three corpora. The frequency of the *negated participants* type ranges between 10% and 17%, with a micro average of 11%. This is the second most prevalent type in BioNLP’09 ST and BioInfer and the third most prevalent type in GENIA. On average, 6% of negated events are of the *negated attribute* type; however, the frequency within the different corpora ranges between 2% and 7%. We note that the BioInfer corpus does not mark temporal or spatial attributes of bio-events. Instead, it incorporates specialized event-types for capturing this type of information. Finally, the *comparisons and contrasts* type accounts for 8% of negated bio-events.

#### Discussion

Previous work on the identification of negated events has primarily been focussed on *negated trigger* events, i.e., the cases where a negation cue modifies the event-trigger. However, our analysis shows that a significant proportion (37%) of negated events belongs to the other types. Therefore, a system for effectively identifying negated bio-events should have the ability to recognise all types of negated events.

The most direct method of facilitating the recognition of a particular negated event-type by the system is to engineer features corresponding to that type, e.g., features based on constituency or dependency relations between the negation cue and the event constituents (triggers, participants and attributes). Since the manifestations of the *comparison and contrast* type usually lack an explicit negation cue, a different approach is required for this type. One possibility would be to identify the comparison/contrast markers and patterns and engineer features based on them. Feature engineering is discussed in detail in the “Feature Design” section below.

### Analysis of negation cues

Although the context and syntactic structure of a sentence play important roles in determining the negation status of a bio-event contained within the sentence, the presence of a negation cue is the most important factor to be considered.

#### Ambiguity of negation cues

Negation cues can be ambiguous [42,53], i.e., in some contexts they may not trigger negations. Wilson et al. [54] pointed out the difference between the lexical and contextual polarities of a word. The **lexical polarity** is the prior or fixed polarity ascribed to a word, based on its meaning and general usage in the language. The **contextual polarity** of a word is more dynamic, and depends on the context of the text fragment containing the word. The contextual polarity can be different from the lexical polarity, and this difference is the key source of ambiguity in determining whether a word or phrase constitutes a negation cue. For example, consider the words *lack* and *loss*. Both of these words have a negative lexical polarity, as they convey the “state of not having something”. That is why they have been identified as negation cues in the BioScope corpus. Morante [42] also identified both of these words as unambiguous negation cues. However, from a biological perspective, these words have a positive polarity when used in the context of a *negative_regulation* event. Hence, a positive contextual polarity can be ascribed to these words in certain instances. Similarly, the words *absent* and *absence* may also be used to convey *negative regulation* rather than negation.

**Figure 8:**
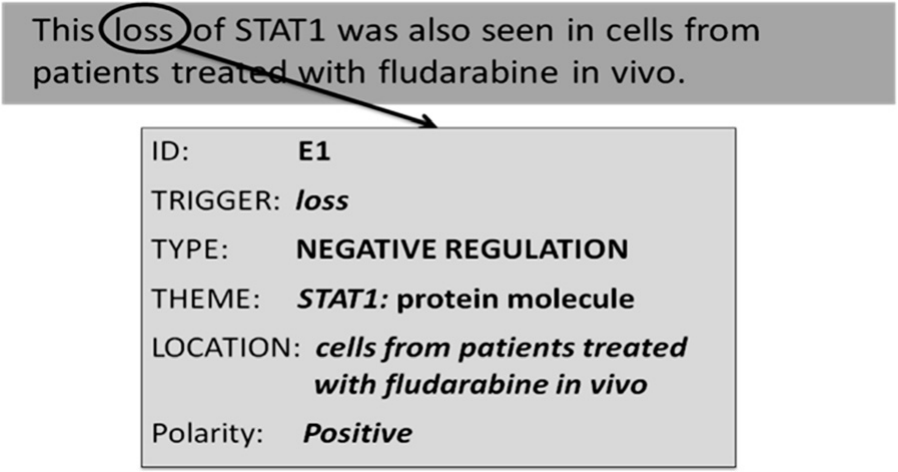
**An instance of the word *loss *with positive contextual (biological) polarity; Source = PMID: 10202937.**

Figure 8 shows a case of conflicting lexical and contextual polarities. In the sentence shown, the event E1 is anchored to the word *loss*, and it expresses the *negative_regulation* of the protein molecule *STAT1* in *cells from patients treated with fludrabine in vivo*. Note that the polarity of E1 is positive.

Based on our analysis of negated bio-events, we conclude that the ambiguity status of a negation cue is not universal. Rather, it is determined by the:

• nature of text under consideration

• annotation perspective (e.g., linguistic or biological)

• context of the surrounding text and the lexical polarity of the cue

#### Indicators of low manner of interaction

Sometimes, the text containing a bio-event also contains a word or phrase that provides an indication of the rate, level, strength or intensity of the interaction. In [55], we refer to this indication as the *manner* of the event, and three types of manner are distinguished: *high*, *neutral* and *low*. The words indicating a low manner include adjectives and adverbs like *weak*, *weakly*, *slight*, *slightly*, *slow*, *small*, *little*, *low*, etc.

**Figure 9:**
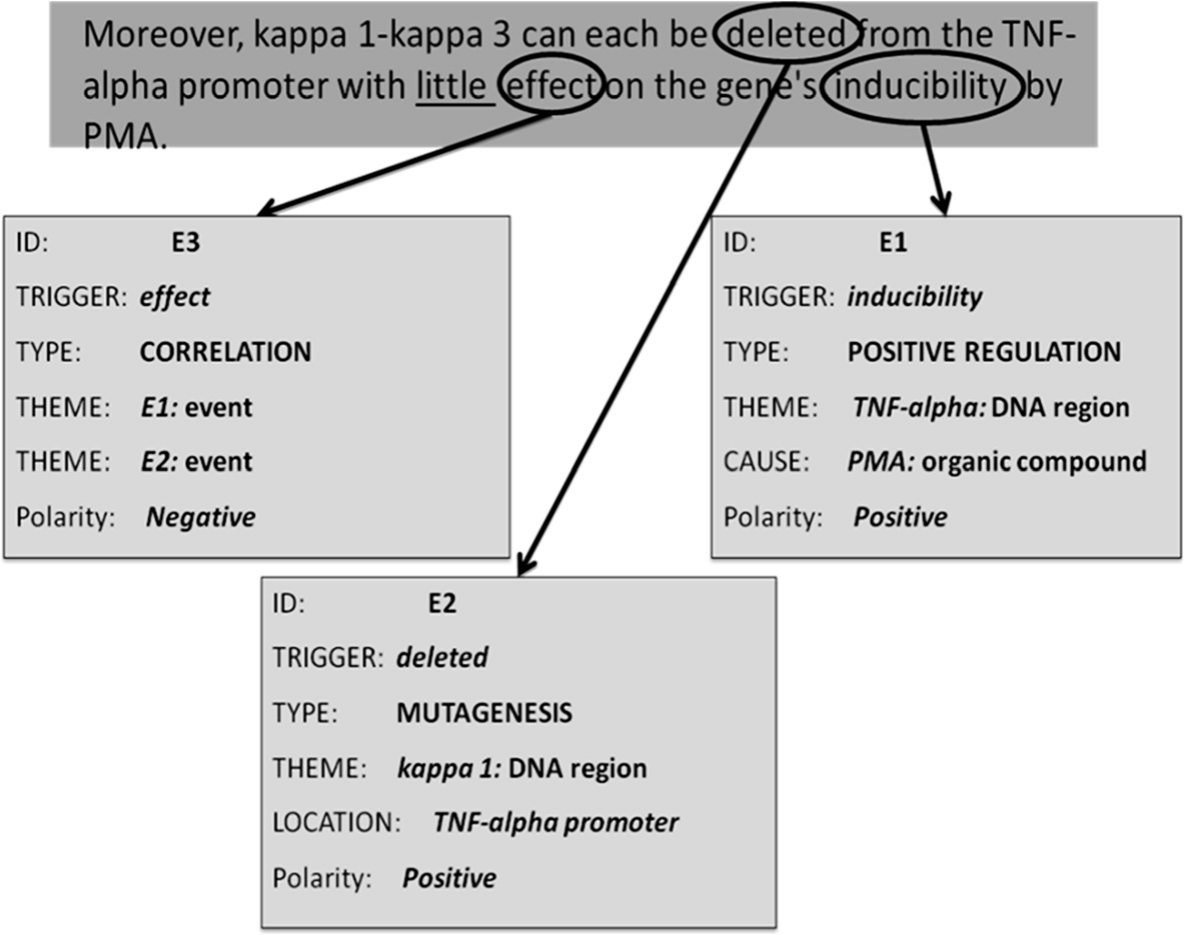
**An instance of the low manner indicator *little *being treated as a negation cue; Source = PMID: 20562282.**

Indicators of low manner have historically been treated as negation cues. In the field of sentiment analysis, such indicators have been considered as a special class of negative polarity indicators. Wiegand et al. [53] refer to this class of cues as *diminishers*, while Wilson et al. [54] have labelled them as *negative polarity shifters*. Similarly, indicators of low manner have been treated as negation cues in the field of biomedical text mining. Examples can be found in the three corpora of negated bio-events (i.e., GENIA Event, BioInfer and BioNLP’09 ST), as well as in the BioScope corpus. Figure 9 shows an example sentence where the low manner indicator *little* has been interpreted as a negation cue for the event E3.

In [55], we proposed an alternative approach to event interpretation. We argued that polarity and manner should be treated as orthogonal dimensions of event interpretation, i.e., the value of manner should not influence the value of polarity and vice-versa. According to this approach, the event E3 in Figure 9 would have a *low* manner but a *positive* polarity.

#### Deactivators of negation cues

The ability of some words to act as negation cues is affected by the syntactic constructions in which they are used. This means that a word that normally acts as a negation cue can cease to act in that capacity if it is preceded and/or followed by certain other words. We refer to these syntactic patterns as *negation deactivation patterns*. Here, we focus only on the two most common negation cues i.e., *no* and *not*.

• Deactivators of not: The word *not* is the most frequent negation cue in the BioScope corpus and accounts for over 41% of the total negation instances in the corpus. However, in almost 8% of cases, it does not indicate a negation, i.e., it ceases to act as a negation cue. In our analysis, we focussed on a simple deactivation pattern: *not < deactivatorOfNot>*. The pattern indicates an occurrence of the word *not* immediately followed by one of its *deactivators*. We only considered the following five deactivators: *clear*, *evident*, *known*, *necessarily* and *only*.

In our analysis of the GENIA Event corpus, we discovered a total of 261 events which belonged to the sentences containing the above pattern. Amongst these, 258 events (99%) were positive and only 3 events (1%) were negated, suggesting that this is an effective pattern for identifying the deactivated instances of the word *not*.

• Deactivators of no: The word *no* is the second most frequent negation cue in the BioScope corpus and accounts for almost 30% of the total negation instances in the corpus. However, in over 6% of cases, it does not indicate a negation. Morante [42] has identified several constructions which contain the word *no*, but do not trigger a negation. These constructions include: *no sign of*, *no evidence of*, *no proof* and *no guarantee that*.

**Figure 10:**
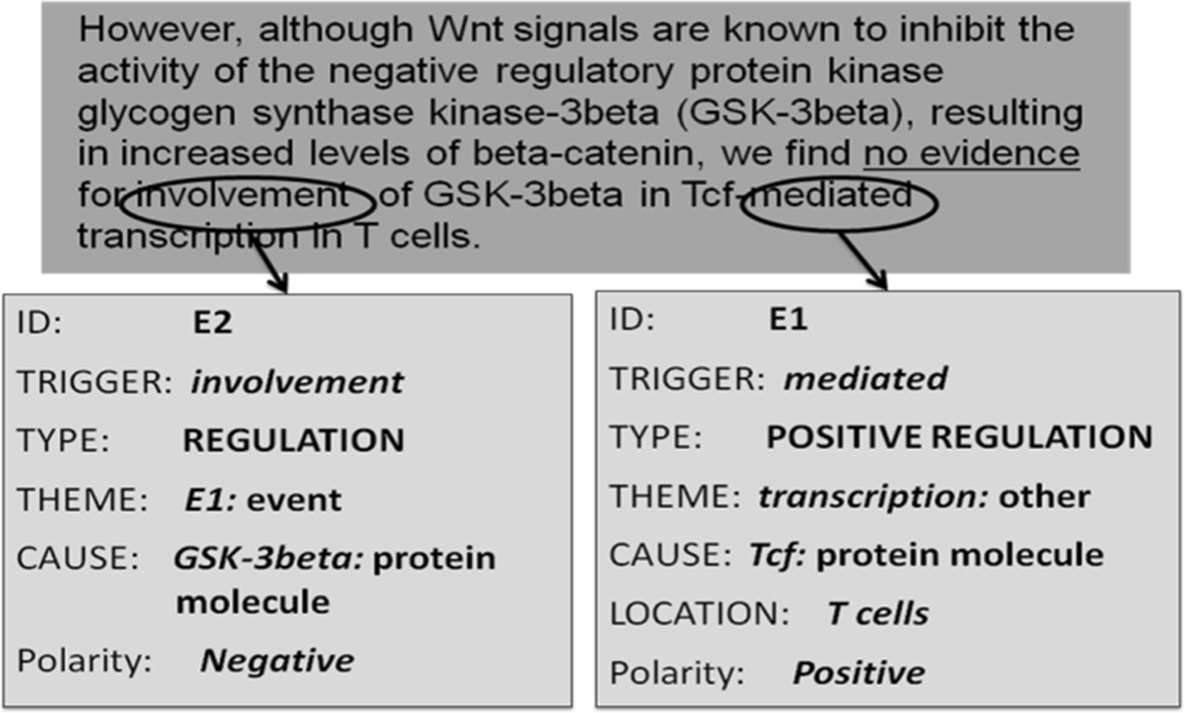
**An instance of negation triggered by the construction *no evidence*; Source = PMID: 10221643.**

Our analysis of the GENIA Event corpus revealed that in some cases, these constructions do trigger negated events. For example, consider the sentence in Figure 10, where the construction *no evidence* triggers the negation of event E2. Based on our analysis, we conclude that the deactivation patterns identified for linguistic (scope) annotation may not hold for biological (event) annotation.

#### Relationship between the negation cues and event-types

We investigated the relationship between negation cues and different types of bio-events. Our analysis revealed two classes of negation cues with respect to event-types. These are:

• Type-independent Negation Cues: This class includes typical negation markers like *no*, *not* and *fail* etc. Some inherently negative event-triggers which can be applied to various types of events are also included in this category. For example, event-triggers like *unaffected* and *independent* can be used for various types of events including *positive_regulation*, *negative_regulation* and *correlation* events.

• Type-dependent Negation Cues: This class includes cues like *immobilize*, *decoupling* and *dysregulation*, which act as negation cues for specific event-types: *immobilize* and *decoupling* are used only for *localization* events, while *dysregulation* is used only for *regulation* events.

#### Corpus / domain idiosyncrasies

Certain cues which are unambiguous and/or frequent in one corpus can be ambiguous and/or scarce in another. For example, words like *protected* and *abolish* are treated as negation cues in BioInfer. However, they are mostly interpreted as indicators of *negative_regulation*, rather than negation, in the GENIA Event and BioNLP’09 ST corpora.

On the other hand, the verb *fail* is frequent and mostly unambiguous as a negation cue in the GENIA Event and BioNLP’09 ST corpora. However, in the BioInfer corpus, it does not appear as a negation cue even once.

#### Compilation of cue lists

Having identified negation cues as an important factor in the identification of negated bio-events, we conclude that it is important to:

• determine the impact of the choice of cue lists on the overall task performance

• identify an optimum cue list for the task

Based on the above analysis, we decided: (1) not to create separate lists for ambiguous and unambiguous cues, (2) to treat *low manner* indicators as negation cues. We then compiled four separate lists of negation cues for comparison. Table 3 depicts the elements in each list. A brief description of each list is as follows:

•c40: We formulated a list of 40 cue words by combining the previously published lists and cues discovered during our own initial analysis of negated bio-events.

•cBioInfer: We extracted negation cues from the BioInfer corpus. This was a straightforward task, because the cues had already been annotated. We discarded the cues which occurred only once in the corpus and labelled the remaining list as cBioInfer.

•cBioScope: This is the list of 28 negation cues, compiled by Morante [42] from the BioScope corpus.

•cCore: We analysed 1,000 randomly selected negated bio-events from the three corpora containing negated bio-events. We recorded all the negation cues observed in these bio-events, and selected the 20 most frequent cues to form this list.

**Table 3:** Negation cue lists

**Name**	**Size**	**Elements**
c40	40	absence, absent, barely, cannot, deficiency, deficient, except, exception, fail, failure, impair, inability, inactive, independent, independently, insensitive, instead, insufficient, lack (noun), lack (verb), limited, little, loss, lose, lost, low, negative, neither, never, no, none, nor, not, prevent, resistance, resistant, unable, unaffected, unchanged, without
cBioScope	28	absence, absent, cannot, could not, either, except, exclude, fail, failure, favor over, impossible, instead of, lack (noun), lack (verb), loss, miss, negative, neither, never, no, no longer, none, not, rather than, rule out, unable, with the exception of, without
cBioInfer	25	abolished, absence, cannot, defective, deficient, despite, differ, different, differential, distinct, failure, independent, independently, lack, negligible, neither, no, nor, not, protected, separately, simultaneously, unable, unlike, without
cCore	20	absence, fail, inability, independent, independently, insensitive, insufficient, lack (noun), lack (verb), little, neither, no, nor, not, resistant, unable, unaffected, unchanged, without

### Feature design

Feature engineering and selection is a vital part of any machine learning system. Various types of features have previously been used for different negation detection tasks. However, most previous work has concentrated on event-triggers, whilst the other semantic aspects of the event (like location and participants) have been ignored. Based on our analysis of negated bio-events, we engineered various syntactic, semantic, lexical, lexico-syntactic and lexico-semantic features. We used the Enju parser [56] for extracting the part of speech (POS) tags, phrase structure trees and the dependency relations. A brief explanation of each feature set is as follows:

• Syntactic Features: These include the POS tags of the event-trigger, event-themes, event-causes and the negation cues found in the sentence.

• Semantic Features: These features are constructed from the semantic information available for the bio-event. They include the semantic type of the bio-event (e.g., *gene_expression*, *localization*, *positive_regulation* etc.), the semantic type of each participant (e.g., *lipid*, *DNA molecule* and *protein complex* etc.) and the role of each participant (e.g., *theme* and *cause*, etc.). We have also used a *complexity* feature, which indicates whether a bio-event is complex, i.e., whether it has one or more participants which are bio-events themselves.

• Lexical Features: These include: whether there is a negation cue present in the sentence, the cue itself, whether a negation deactivator is present and its relative position with respect to the negation cue.

• Lexico-Semantic Features: These features are constructed using a combination of the “textual” bio-event information and the sentence containing the bio-event. The textual bio-event information includes the text fragment indicating the occurrence of the bio-event (i.e., the event-trigger), the text fragments identifying the event participants and the text fragments indicating any event attributes, like location, etc. These features also include the surface distances between the negation cue and the event-trigger and event-location, whether the negation cue is part of the event-trigger and whether the negation cue precedes or follows the event-trigger.

• Dependency (Lexico-Syntactic) Features: These features are constructed using the textual bio-event information and the dependency relations found in the sentence. They include: whether direct and/or indirect dependency relations exist between the negation cue and the event-trigger and/or event-location, the types of these dependency relations and the length of the dependency chains.

• Constituency (Lexico-Syntactic) Features: These include *command* and *scope* features. The concept of a *command relation* was first introduced by Langacker [57] as a means of identifying the nodes affected by a given element in the constituency parse tree of a sentence. He defined an S-command relation as follows: ‘a node *X* commands a node *Y* if neither *X* nor *Y* dominates the other and the *S* (sentence) node most immediately dominating *X* also dominates *Y*’. We used several command features including the existence of S-, VP- and NP-command relations between the negation cue and the event-trigger and/or event-location. The scope features were engineered using the information pertaining to whether the event-trigger, event-participants or event-location fall under the syntactic scope of the negation cue.

### Machine learning algorithms

The choice of machine learning algorithm can significantly influence the performance of a classification task. This has been demonstrated for various natural language processing tasks including text categorisation [58], word sense disambiguation [59] and the detection of negated terms [48]. In order to measure the impact of the choice of learning algorithm on the task of identifying negated bio-events, we decided to compare the performance of the most commonly used learning algorithms. We chose the following six algorithms, and used their WEKA [60] library implementations to carry out our experiments:

•**Decision Trees:** Decision Tree algorithms learn rules that are expressed as “conjunctions of constraints on the attribute values of instances. Each path from the tree root to a leaf corresponds to a conjunction of attribute tests and the tree itself to a disjunction of these conjunctions” [61]. Various decision tree algorithms have been proposed over the years. However, we concentrated on C4.5 [62], which is an enhanced version of ID3 [63]. The C4.5 algorithm constructs the decision tree by choosing the attribute with the highest value of normalised information gain at each node and creates new branches corresponding to the different values of this attribute. Once the initial tree has been created, the algorithm tries to identify and remove the least useful branches. Decision trees have been used extensively for various problems in bioinformatics [64]. They have also been used to detect negations in medical texts [47]. Our implementation of C4.5 used the following optimisation settings: (1) apply sub-tree replacement, (2) apply sub-tree raising, (3) require a minimum of 2 instances per leaf, (4) set a confidence threshold for pruning of 0.25.

• Random Forest: The Random Forest [65] algorithm develops an ensemble (i.e., a forest) of decision trees from randomly sampled subspaces of the input features. Once the forest has been created, new objects are classified using a two-step process: (1) An individual classification is obtained from each tree in the forest, (2) The final classification of the object is determined by majority votes among the classes obtained from individual trees. Despite being successfully used for various text mining and bioinformatics tasks [66,67], the Random Forest algorithm has not been previously used for detecting negation scopes, negated concepts or negated events. Our implementation of Random Forest used the following optimisation settings: (1) set the number of trees in the forest to 10, (2) set the number of features used to build individual trees to log(N + 1), where N is the total number of features, (3) set no restrictions on the depth of individual trees.

• Logistic Regression: Logistic Regression classifiers try to predict the class probability of an object by fitting the training data to a logistic function [61]. Logistic Regression classifiers have previously been used to identify negated bio-events [31].

• Naïve Bayes: Naïve Bayes is one of the simplest probabilistic classification algorithms. It uses the Bayes probability model for predicting the class probabilities of inputs. The word *naïve* indicates that the algorithm assumes class conditional independence, i.e., it assumes that the effect of a variable value on a given class is independent of the values of other variables [61]. Despite its simplicity, the Naïve Bayes algorithm achieves good results for many complex classification problems [68]. It has also been used to detect negations in medical texts [47,48]. Our implementation of Naïve Bayes used a default precision of 0.1 for numeric attributes for cases of zero training instances.

• SVM: Support Vector Machines (SVMs) [69] perform classification by constructing an *N*-dimensional hyperplane that optimally separates the data into two categories. They use a kernel function to transform the data into a higher dimensional space, which paves the way for optimal separation. Many previous studies in negation detection have used SVMs [33,43,48]. Our implementation of SVM replaced all missing values, and converted the nominal attributes to binary attributes. It also normalised all attributes by default. We used: (1) a polynomial kernel, (2) the default value of the complexity constant.

• Instance-Based Algorithms: Instance-Based (also known as Memory-Based) learning algorithms do not derive generalisations or abstractions from the complete training data. Rather, they keep all training data in memory, and generate classification predictions using only the most similar training instances. IB1 [70] is an instance-based learning algorithm. It uses normalised Euclidean distance to find the training instance closest to a given test instance, and predicts the same class as this training instance. IB1 is similar to the nearest neighbour algorithm, except that it normalises its attributes’ ranges, processes instances incrementally, and has a simple policy for tolerating missing values. Instance-based learning algorithms have previously been used for detecting negation cues and their scopes [43].

## Results

We ran a series of experiments to obtain the best results for each dataset and to systematically evaluate the impact of using different cue lists and learning algorithms. This section describes the results of our experiments. All results are based on 10-fold cross validation. We have used the standard metrics of precision, recall and F-score for reporting and comparing results. Precision is the number of true positives divided by the sum of true positives and false positives; recall is the number of true positives divided by the sum of true positives and false negatives; and F-score is the first harmonic mean of precision and recall.

### Best results for each dataset

On the GENIA Event dataset, the best results were achieved by the Random Forest classifier using the c40 cue list. The classifier achieved 83% precision and 67% recall, which equates to an F-score of 74%. The same classifier achieved the best results on the BioNLP’09 ST dataset, achieving approximately 78% precision, 64% recall and 70% F-score. The best results on the BioInfer dataset were also achieved by a Random Forest classifier. However, the cBioInfer cue list was used to engineer the features. This classifier achieved 86% precision, 85% recall and 85% F-score. Table 4 shows the best results achieved for each dataset.

**Table 4:** Best results for each dataset

**Dataset**	**Precision**	**Recall**	**F-Score**	**Algorithm**	**Cue list**
GENIA Event	83.1%	67.1%	74.2%	Random Forest	c40
BioInfer	86.1%	84.5%	85.3%	Random Forest	cBioInfer
BioNLP’09 ST	77.6%	63.9%	70.1%	Random Forest	c40

### Cue list comparison

In order to compare the performance of the four cue lists, we ran a series of experiments using each cue list (in turn) to engineer the features. In all cases, the Random Forest algorithm was used, as it had consistently produced the best results for all datasets. Table 5 shows the performance of the classifiers trained using the four cue lists for each of the datasets. The key results are as follows:

• The use of the c40 cue list resulted in high performance on all three datasets. This list resulted in better performance than other cue lists on GENIA Event and BioNLP’09 ST, achieving the highest precision and recall on both datasets. However, on BioInfer it resulted in lower performance than when the cBioInfer and cCore lists were used.

• The use of the cCore cue list resulted in consistent performance, achieving the second best results (F-score) for all three datasets. The results were very close to the top performing cue list for GENIA Event and BioNLP’09 ST, with margins of 0.5% and 1.8%, respectively. However, on BioInfer, the F-score was 7% less than when the cBioInfer list was used.

• Using the cBioInfer cue list caused a significant performance drop on GENIA Event and BioNLP’09 ST (by almost 5% and 8%, respectively), compared to when c40 and cCore were used. However, as expected, it resulted in the best performance on BioInfer by a fair margin (over 7%).

• The classifiers trained using the cBioScope cue list achieved the lowest results for all three datasets by significant margins (ranging between 6% and 8%).

**Table 5:** Comparison of results using different cue lists

**Cue List**	**GENIA event**			**BioInfer**			**BioNLP’09 ST**		
	**P**	**R**	**F**	**P**	**R**	**F**	**P**	**R**	**F**
c40	**83.1**%	**67.1**%	**74.2**%	84.4%	70.8%	77.0%	**77.6**%	**63.9**%	**70.1**%
cCore	82.6%	66.7%	73.8%	87.0%	70.8%	78.1%	76.3%	61.6%	68.2%
cBioInfer	81.4%	60.4%	69.3%	86.1%	**84.5**%	**85.3**%	75.3%	53.2%	62.3%
cBioScope	80.7%	59.9%	68.8%	**89.3**%	67.7%	77.0%	75.4%	52.9%	62.2%

**Figure 11:**
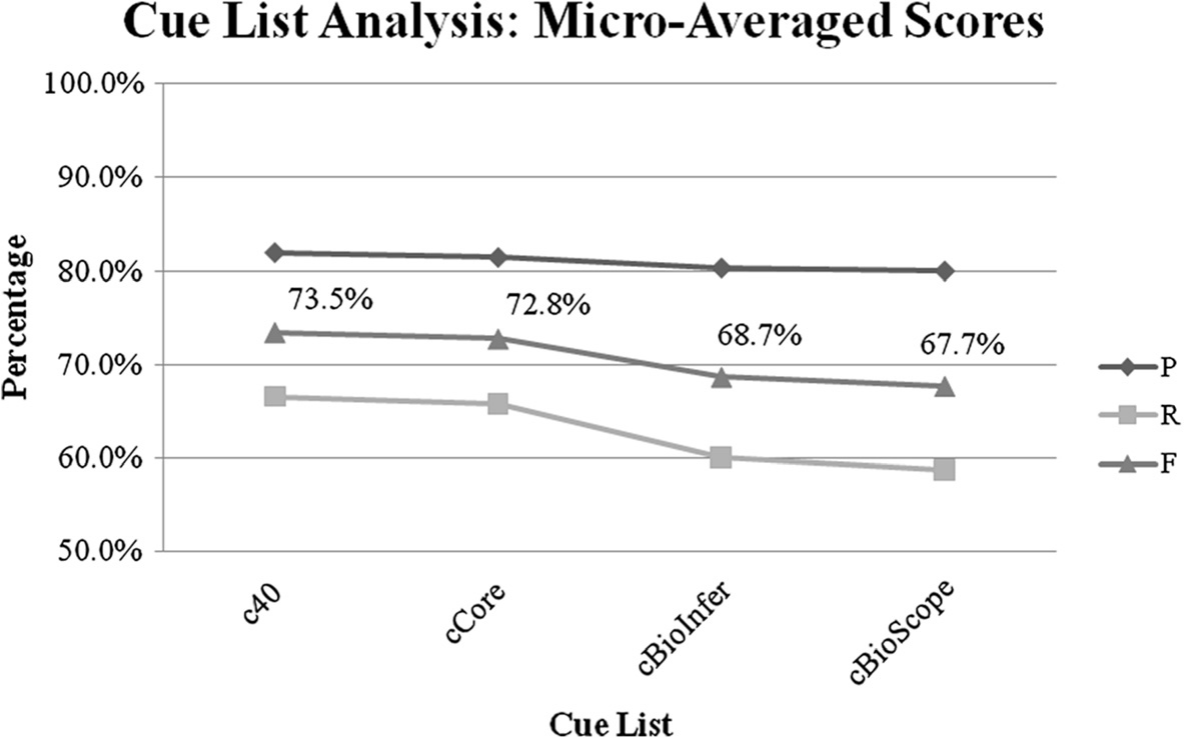
Cue list comparison: Micro-averaged results for the three datasets.

Figure 11 shows the micro-averaged results for each cue list. It shows that overall (in terms of F-score), c40 performed the best, followed by cCore (−0.7%), cBioInfer (−4.8%) and cBioScope (−5.7%), respectively.

The difference between the best and the worst performance caused by the choice of cue list was 5% for GENIA Event, 7% for BioInfer and 8% for BioNLP’09 ST. This provides sufficient evidence in favour of the hypothesis that the choice of the negation cues used for engineering the feature set has a significant impact on the performance of a system designed for the identification of the negated bio-events.

### Algorithm comparison

In order to compare the performance of the chosen learning algorithms for the task of identifying negated bio-events, we ran a series of experiments on each dataset. In each experiment, we constructed a classifier using the chosen algorithm. The features were engineered from the cCore cue list. We chose the cCore cue list because it had performed consistently on all three datasets. Table 6 shows the results for each dataset. The key findings are as follows:

• C4.5 performed consistently (in terms of F-score) on all three datasets. It outperformed the other algorithms on BioNLP’09 ST, scored second on GENIA Event and fourth on BioInfer.

• Random Forest outperformed the other algorithms on GENIA Event and BioInfer, and scored second on the BioNLP’09 ST by a narrow margin of 0.8%.

• Logistic Regression achieved the third best results on both GENIA Event and BioInfer. It scored fourth on BioNLP’09 ST.

• Naïve Bayes achieved the highest recall for all datasets. However, its precision was noticeably low (ranging between 32% and 42%), which led to the lowest F-scores for all datasets.

• SVM scored fifth for all three datasets. Although it performed much better than Naïve Bayes, it was significantly behind Random Forest and C4.5.

• IB1 produced the second best results for BioInfer and the fourth best results for both GENIA Event and BioNLP’09 ST.

**Table 6:** Comparison of results using different learning algorithms

**Algorithm**	**GENIA event**			**BioInfer**			**BioNLP'09 ST**		
	**P**	**R**	**F**	**P**	**R**	**F**	**P**	**R**	**F**
C4.5	**84.4**%	62.4%	71.8%	82.1%	68.3%	74.6%	**82.2**%	56.5%	**67.0**%
Random Forest	82.6%	66.7%	**73.8**%	**87.0**%	70.8%	**78.1**%	76.3%	58.4%	66.2%
Logistic Regression	82.8%	58.7%	68.7%	79.3%	71.4%	75.1%	80.5%	53.1%	64.0%
Naïve Bayes	31.6%	**83.0**%	45.8%	42.2%	**83.9**%	56.2%	32.9%	**82.3**%	47.0%
SVM	79.3%	53.7%	64.0%	79.0%	67.7%	72.9%	78.6%	46.7%	58.6%
IB1	66.1%	66.7%	66.4%	85.8%	71.4%	77.9%	70.8%	59.5%	64.7%

**Figure 12:**
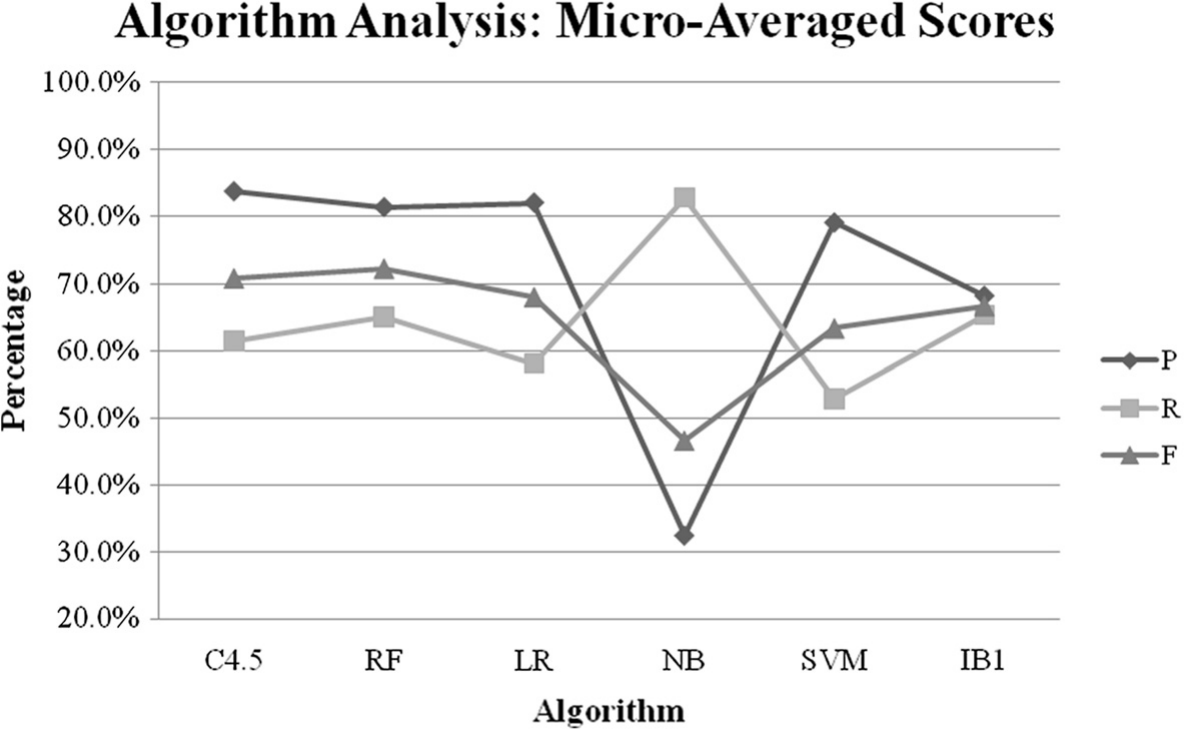
Algorithm comparison: Micro-averaged results for the three datasets.

Figure 12 shows the micro-averaged results for each algorithm. It shows that overall (in terms of F-score), Random Forest performed the best, followed by C4.5 (−1.5%), Logistic Regression (−4.3%), IB1 (−5.6%), SVM (−8.9%) and Naïve Bayes (−25.7%).

The difference between the best and the worst performing algorithms was 28% for GENIA Event, 22% for BioInfer and 20% for BioNLP’09 ST. Even if we exclude Naïve Bayes, which performed significantly worse than the rest of the algorithms, the difference was still 10% for GENIA Event, 5% for BioInfer and 8% for BioNLP’09 ST. This provides sufficient evidence in favour of the hypothesis that in addition to the choice of negation cue list, the choice of learning algorithm also has a significant impact on the performance of a (machine learning) system for identifying negated bio-events.

## Discussion

### Comparison with previous results

As mentioned earlier, the identification of negated bio-events is a new area of research and only a few results have been reported previously. The best results on the identification of negated bio-events were reported by Sarafraz and Nenadic [33]. They used the *Training* subset of the BioNLP’09 ST dataset for training their system and the *Development* subset for testing. They achieved 38% precision, 76% recall and 51% F-score. In comparison, our system achieved an F-score of above 70% with 10-fold cross validation on the entire BioNLP’09 ST dataset. In order to obtain a more direct comparison, we conducted further experiments with the same experimental settings as those used by Sarafraz and Nenadic. That is, we trained our Random Forest classifier on the *Training* subset of the BioNLP’09 ST data and tested it on the *Development* subset. In this setting, our system achieved an F-score of just under 70%, which is significantly higher than the results of Sarafraz and Nenadic.

Our system achieved even better results on the GENIA Event (74% F-score) and BioInfer (85% F-score) datasets. This is particularly encouraging, as these corpora contain more complex and varied bio-events than the BioNLP’09 ST corpus.

### Selection of negation cues

Various lists of negation cues have previously been proposed for different negation detection tasks. With respect to the task of identifying negated bio-events, the main questions about the nature, role and processing of negation cues are as follows:

• Does a “universal” list of negation cues exist? Our analysis of negated bio-events confirmed that negation cues are ambiguous. Whether a word acts as a negation cue for a bio-event depends on the lexical as well as the contextual polarity of the word. While the lexical polarity of a word remains fixed, its contextual polarity depends on a number of factors, including the nature/domain of the text, the annotation perspective, the context and the syntactic structure of the sentence. Therefore, it is hard to compile a universal list of negation cues. However, the potential utility of domain specific lists has been reinforced by our experiments. The c40 and cCore cue lists showed consistently high performance across the three bio-event corpora.

• What is the impact of the choice of a negation cue list on the overall system performance? We designed experiments to measure the impact of the choice of a negation cue list on the overall system performance. We found that a significant variation (ranging between 5% and 8%, depending on the corpus) in the system performance was caused by the cue list used.

• Should negation cues be annotated in gold standard corpora? BioInfer is the only corpus of bio-events containing annotation of negation cues. We compiled a list of negation cues identified in the corpus, and labelled it cBioInfer. The use of the cue list did not result in high performance when applied to the other two datasets (i.e., GENIA Event and BioNLP’09 ST). However, its use on the BioInfer dataset resulted in better performance than when other cue lists were used, by a significant margin of 7%. These results provide strong evidence that both event and corpus characteristics, as well domain, can determine the most appropriate set of negation cues to use in a classifier. Thus, although some sets of domain specific cue lists (e.g., c40 and cCore) can provide consistent performance across different corpora, explicit annotation of negation cues in different gold standard corpora will allow further sets of cue lists to be produced. These lists will be tuned not only to the domain, but also to different types of event specifications. These findings favour the wider argument that we made in Nawaz et al. [55] for the annotation of lexical cues indicating different aspects of the correct interpretation of an event.

### Feature engineering and selection

In comparison to previous work, our feature engineering approach has the following unique aspects:

• use of a combination of syntactic, lexical, semantic, lexico-semantic and lexico-syntactic features

• incorporation of all available textual fragments associated with the bio-event (including the trigger, participants and attributes of the event)

• incorporation of event hierarchy information (i.e., complexity status)

• incorporation of negation deactivators

• incorporation of constituency as well as dependency relations/scopes.

We conducted additional experiments to evaluate the relative performance and contribution of the different types of features and their combinations. We were particularly interested in the comparison of the dependency and the constituency features, as both have previously been used for the task of identifying negated bio-events. Kilicoglu and Bergler [30] used a rule-based approach based on dependency relations between the negation cues and the event-triggers, whilst MacKinlay et al. [31] used features derived from the dependency parse of the sentence containing the bio-event. However, Sarafraz and Nenadic [33] used command features to achieve better performance.

The evaluation of the individual feature sets showed that dependency and lexical features achieved results more than twice as high as command features. Similarly, the combination of lexical and dependency features achieved significantly better results than the combination of the lexical and command features. Based on these results, we conclude that, in terms of individual contribution, dependency features outperform constituency features by a significant margin. This is consistent with the previously reported comparisons between dependency and constituency features for the tasks of opinion mining [71,72] and PPI extraction [73]. However, it is important to emphasise that our conclusions are based on specific representations of constituency features that we have used in our experiments. It would be interesting to explore and compare other representations of constituency features for this task.

We also observed that the features based on the POS tags of negation cues, event-triggers, event-themes and event-causes did not improve the performance. Similarly, the features based on the semantic types of event-themes and event-causes did not influence the performance either. This suggests that the polarity status of a bio-event is influenced neither by the semantic types of its participants nor by the POS tags of text fragments associated with the event.

### Algorithm selection

We designed a series of experiments to evaluate and compare the performance of six learning algorithms with respect to the task of identifying negated bio-events. All of these algorithms, with the exception of Random Forest, had previously been used for different negation detection tasks with varying degrees of success. Our results showed that, on average, the Random Forest algorithm performs the best, while the Naïve Bayes algorithm achieves the lowest results by a huge (26%) margin.

Our results are consistent with Caruana and Niculescu-Mizil [74], who conducted a wide-ranging study, comparing the performance of ten supervised learning methods. They measured the performance of each method on 11 different binary classification problems, and found that Random Forest outperformed the other algorithms. Our results are also consistent with Goryachev et al. [48], who compared the performance of SVM and Naïve Bayes for the task of detecting negations in medical texts. They found that SVM outperformed Naïve Bayes by a significant margin (8%). On the other hand, Goldin and Chapman [47] compared the performance of Naive Bayes and decision trees for the task of identifying negated terms in medical texts. They found that Naïve Bayes outperforms decision trees by a small (1%) margin. Similarly, for the task of identifying negation scopes in biomedical research literature, Morante and Daelemans [43] obtained analogous results for Instance-Based learning and SVM. In contrast to these results, we found that Naïve Bayes performs significantly worse than decision trees, and that Instance-Based learning outperforms SVM. This contrast shows that different learning algorithms do not perform consistently for different negation detection tasks. This leads us to the following conclusions:

• Despite the apparent similarities, the task of identifying negated bio-events is inherently different from the other negation detection tasks like negated term detection and negation scope detection.

• Since the Random Forest algorithm clearly outperforms the other learning algorithms for the task of identifying negated bio-events, its feasibility for other negation detection tasks should be investigated.

### Effect of corpus size

We used all three open access corpora of negated bio-events in our experiments. Table 1 shows the statistics for these corpora. The GENIA Event corpus is the largest and contains bio-events of 36 different semantic types. The BioNLP’09 ST corpus contains only 9 types of bio-events, and it is over three times smaller than the GENIA Event corpus. The best results (10-fold cross validation) achieved on the BioNLP’09 ST corpus were 4% less than the best results achieved on the GENIA Event corpus. The BioInfer corpus is the smallest in size (almost 14 times smaller than GENIA Event) and the most complex, with 60 different event types. Despite these factors, consistently better results were achieved on BioInfer, irrespective of the cue list used. This suggests that there is not necessarily a close correlation between the size of the corpus used for training and the overall performance of the system. We further tested this hypothesis by conducting an additional experiment on the GENIA Event corpus. Instead of performing 10-fold cross validation, we trained the classifier using only half the instances and tested on the other half. We repeated this experiment ten times with randomly selected training and testing datasets, and the average F-score was only slightly (0.5%) less than the F-score achieved by the 10-fold cross validation. Although it goes without saying that very small corpora would not be effective for training, our results suggest that it is not necessarily the case that the larger the corpus used for training, the better the results will be. Rather, the specific features of the annotated events appear to have more of an impact on the performance. In general, it seems that the level of detail of the information annotated for each event, in particular the text fragments associated with the event, is more important than the corpus size. The relatively poor performance achieved on the BioNLP’09 ST corpus could also be explained by the fact that both GENIA Event and BioInfer contain more information about the location of the events than BioNLP’09 ST.

### Correlation between event-type and polarity

Our analysis of negated bio-events revealed that certain words act as negation cues only in the context of specific types of events (see section 5.1.4). Apart from this, we did not find any evidence of “linguistic correlation” between the semantic type of an event and its polarity. However, we did find some “statistical correlation” between event-type and polarity. For example, in the BioNLP’09 ST corpus, 9% of *regulation* events are negated, whereas only 5% of *binding* events are negated. Based on this observation, we engineered two semantic features: one based on the event-type and the other on its complexity status (i.e., whether the event is simple or complex). Both of these features scored low gain ratios on all three datasets. However, the addition of these features improved the overall performance by 0.5% to 1%, depending on the dataset.

In order to further investigate the correlation between event-type and polarity, we designed two experiments:

• Three-Way Splitting: This experiment was similar to the one reported by Sarafraz and Nenadic [33]. The bio-events in the BioNLP’09 ST dataset were split into three classes. The *localization*, *transcription*, *protein_catabolism*, *gene_expression* and *phosphorylation* events were grouped together as *Class-1*. The *binding* events were grouped as *Class-2*, and the *regulation* events (*regulation*, *positive_regulation* and *negative_regulation*) were grouped together as *Class-3*. The Random Forest classifier was trained and tested for each class, separately. The micro averages for precision, recall and F-score were used to measure the overall performance. In comparison to the results achieved without data splitting, the three-way splitting model showed a considerable (21%) improvement in precision. However, the recall dropped significantly (15%), causing an F-score decrease of almost 2%. This is in contrast to Sarafraz and Nenadic, who achieved an increase in both recall and precision. In terms of individual classes, *Class-3* and *Class-1* achieved results which were slightly higher and slightly lower than the single-class model, respectively. However, *Class-2* scored significantly (29%) worse. We experimented with various algorithms and cue-lists, but we were not able to improve the performance for class-2 by more than 2%.

• Two-Way Splitting: In this experiment, we split the bio-events according to their complexity status, i.e., *simple* or *complex*. We performed the two-way splitting on the BioNLP’09 ST data, then trained and tested our Random Forest classifier separately for each class. The results were even worse than the three-way splitting model, and an overall (micro-averaged) performance loss of 5% was observed. In order to test the concept further, we repeated the two-way splitting experiment with the GENIA Event corpus. Again, we observed a significant (4%) decrease in performance. In terms of individual classes, the *complex* class performed better than the *simple* class. We further experimented with various algorithms and cue-lists, but we were not able to improve the performance on the *simple* class by more than 1%. We also observed that over 10% of *complex* events are negated, whereas only 4% of *simple* events are negated. Therefore, a *complex* event is 2.5 times more likely to be negated than a *simple* event.

These experiments show that there is some correlation between the event-type and polarity. However, designing individual classifiers for different types of events does not improve the overall system performance. The classification performance improves for certain classes of bio-events (e.g., *complex* event and *regulation* events), and deteriorates for certain other classes (e.g., *binding* and *simple* events). This variation in performance is mainly due to an uneven distribution of negated bio-events across these classes.

## Conclusion

We have conducted a detailed analysis of the problem of identifying negated bio-events, given gold standard event annotations. We examined the types of negation in three open access corpora of negated bio-events (i.e., GENIA Event, BioInfer and BioNLP’09 ST), and identified five main types of negated bio-events, based on the lexico-semantic mechanisms affecting the polarity of an event. Our analysis showed that a significant proportion (37%) of negated bio-events cannot be detected by considering the event-trigger alone. It also revealed that identification of negated bio-events is a complex task that requires a deeper level of analysis than that required for tasks such as negated term detection and negation scope detection. Following our analysis of negated event types, we identified the three key aspects of a machine learning based solution to the problem of negated bio-event detection. These are: the compilation of a negation cue list, the design and selection of suitable features and the choice of machine learning algorithm. In order to analyse these aspects, we conducted a series of experiments on the three bio-event corpora. The results confirmed that each one of the three aspects can have a significant impact on the overall system performance. Our analysis showed that the ability of a word/phrase to act as a negation cue depends not only on the context and domain of text, but also on the annotation/information perspective (e.g., linguistic vs. biological perspective). Therefore, there is a need for domain specific lists of negation cues. We compiled two such lists (c40 and cCore), both of which performed consistently in all experiments. We also discovered that, for the task of identifying negated bio-events, the Random Forest algorithm consistently outperforms five other learning algorithms. Combining the best cue lists, feature sets and learning algorithms, we created a novel framework for the identification of negated bio-events. We evaluated our system on the three open access corpora of negated bio-events mentioned above. Our results on the BioNLP’09 ST corpus were significantly higher than the previously reported best results. We achieved even better results on the GENIA Event and BioInfer corpora, both of which contain more varied and complex events.

As mentioned earlier, our system assumes that event annotation has already been performed. As future work, we plan to integrate our system with the EventMine system [75]. The resulting system will be able to extract bio-events of the specified polarity from plain text documents, and will serve as the foundation for a more elaborate system for detecting textual contradictions. We also intend to use our system for enriching other bio-event corpora (like GREC) with polarity information. Although we have focussed on the identification of negated bio-events, our approach can be modified straightforwardly for events in other textual domains. We also plan to explore this further.

## Competing interests

The authors declare that they have no competing interests.

## Authors’ contributions

All authors contributed to the production of the manuscript. RN conceived of and designed the experiments. PT helped with the production of manuscript, and SA supervised all steps of the work. All authors read and approved the final manuscript.
